# Influence and optimization strategy of the magnetic field in 1.5 T MR-linac liver stereotactic radiotherapy

**DOI:** 10.1186/s13014-023-02356-8

**Published:** 2023-10-04

**Authors:** Xin Liu, Peijun Yin, Tengxiang Li, Yong Yin, Zhenjiang Li

**Affiliations:** 1 Department of Oncology, The Affiliated Hospital of Southwest Medical University, Luzhou, 646000 China; 2 Department of Radiation Physics, Shandong Cancer Hospital and Institute, Shandong First Medical University and Shandong Academy of Medical Sciences, Jinan, 250117 China; 3 School of Nuclear Science and Technology, University of South China, Hengyang, 421001 China

**Keywords:** Liver cancer, Radiotherapy, MR-LINAC, Magnetic field, Field

## Abstract

**Objective:**

To compare intensity reduction plans for liver cancer with or without a magnetic field and optimize field and subfield numbers in the intensity-modulated radiotherapy (IMRT) plans designed for liver masses in different regions.

**Methods:**

This retrospective study included 62 patients who received radiotherapy for liver cancer at Shandong Cancer Hospital. Based on each patient's original individualized intensity-modulated plan (plan_1.5 T_), a magnetic field-free plan (plan_0 T_) and static intensity-modulated plan with four different optimization schemes were redesigned for each patient. The differences in dosimetric parameters among plans were compared.

**Results:**

In the absence of a magnetic field in the first quadrant, PTV D_min_ increased (97.75 ± 17.55 vs. 100.96 ± 22.78)%, D_max_ decreased (121.48 ± 29.68 vs. 119.06 ± 28.52)%, D_98_ increased (101.35 ± 7.42 vs. 109.35 ± 26.52)% and HI decreased (1.14 ± 0.14 vs. 1.05 ± 0.01). In the absence of a magnetic field in the second quadrant, PTV D_min_ increased (84.33 ± 19.74 vs. 89.96 ± 21.23)%, D_max_ decreased (105 ± 25.08 vs. 104.05 ± 24.86)%, and HI decreased (1.04 ± 0.25 vs. 0.99 ± 0.24). In the absence of a magnetic field in the third quadrant, PTV D_max_ decreased (110.21 ± 2.22 vs. 102.31 ± 26)%, L-P V_30_ decreased (10.66 ± 9.19 vs. 5.81 ± 3.22)%, HI decreased (1.09 ± 0.02 vs. 0.98 ± 0.25), and PTV D_min_ decreased (92.12 ± 4.92 vs. 89.1 ± 22.35)%. In the absence of a magnetic field in the fourth quadrant, PTV D_min_ increased (89.78 ± 6.72 vs. 93.04 ± 4.86)%, HI decreased (1.09 ± 0.01 vs. 1.05 ± 0.01) and D_98_ increased (99.82 ± 0.82 vs. 100.54 ± 0.84)%. These were all significant differences. In designing plans for tumors in each liver region, a total number of subfields in the first area of 60, total subfields in the second zone of 80, and total subfields in the third and fourth zones of 60 or 80 can achieve the dose effect without a magnetic field.

**Conclusion:**

In patients with liver cancer, the effect of a magnetic field on the target dose is more significant than that on doses to organs at risk. By controlling the max total number of subfields in different quadrants, the effect of the magnetic field can be greatly reduced or even eliminated.

## Introduction

According to the latest global cancer statistics, the mortality and incidence of liver cancer rank third and sixth in the world, respectively, and liver cancer has the second highest mortality rate among all cancers in China [1]. Because most patients with liver cancer have missed the opportunity for surgery and have a poor prognosis and short survival time, neoadjuvant radiotherapy (RT) plays an important role in the multidisciplinary treatment of advanced liver cancer.

Cone-beam computed tomography (CBCT) image-guided radiotherapy (IGRT) has become one of the methods to determine the exact location of the tumors to be treated. Although CBCT is very effective in the development of RT, it does not provide the best soft tissue contrast, especially for the treatment of tumors in the prostate, brain and abdomen [2, 3]. In recent years, in RT, there has been a trend to study and use magnetic resonance imaging (MRI) as an imaging tool to better display soft tissue and depict tumors more accurately. In cancer radiotherapy, MR image guidance provides many benefits [4], such as depicting target volume [5], helping with motion management [6], and adapting to anatomical changes [7], which are useful for the real-time, high-contrast visualization of tumors and organs at risk (OARs). Nonetheless, despite the benefits of magnetic resonance imaging guidance, it is also important to study the effect of magnetic fields on dose distribution.

In recent years, with the development and clinical application of magnetic resonance linear accelerators (MR-LINAC), people have become increasingly interested in the effect of magnetic fields on dose distribution, and some research results have appeared [8, 9]. The MR-LINAC receives a static magnetic field perpendicular to the beam direction. In the MR-LINAC, the Lorentz force exerted by a static magnetic field makes the secondary electron move perpendicular to its velocity. Inside the body, the Lorentz force reduces the stacking depth and leads to asymmetry of the transverse beam profile [10]. At the tissue-air interface, the magnetic field returns electrons that leave the tissue to the surface, which is called the electron return effect (ERE) [11]. This effect is obvious at interfaces where there is a great difference in density and leads to a significant change in the dose at the interface. Because of this effect, hot spots and cold spots appear around the air cavity. The main concern is that in the presence of air in and near the target, the application of such a magnetic field in RT may affect the dose distribution due to the Lorentz force. According to several research, in patients treated on 1.5 T MR-LINAC, the air cavity in the rectum will alter the measurement of the rectal prescription dose due to ERE, and the size of the air cavity will determine the position and size of hot spots [12]. The effectiveness of MR-linac radiation in the case of anatomical alterations in head and neck cancer was assessed by Chuter et al. The findings demonstrated that neither the planning target volume (PTV) nor the OARs related to head and neck cancer were significantly affected by the magnetic field or the target dose [13]. Heijst et al. studied the effect of a magnetic field on the skin dose in breast radiotherapy and found that the increase in skin dose caused by accelerated local breast irradiation was less than that caused by whole breast irradiation [14]. However, at present, the effect of magnetic fields on the radiation dose for liver cancer is still unclear.

Intensity-modulated radiotherapy (IMRT) is a common clinical method for the treatment of cancer [15]. Compared with traditional RT, IMRT is different in that it reverses the treatment planning process and uses many fields or subfields to provide a high-precision conformal dose distribution [16]. Moreover, the Multi-Leaf Collimator (MLC) is irregular and the beam control ability is stronger [17]. MLCs are used for IMRT to generate the optimal dose model for each treatment area based on the dose constraints set by the PTV and OARs. The liver belongs to the parenchymal organs, which are surrounded by the heart, kidney, lung, stomach, small intestine and other OARs [18]. Due to its unique anatomical structure, ERE affects liver tumors in various ways, and the magnetic field dose of liver tumors that are close to hollow organs is significantly altered. among which there are many hollow organs. Because of the liver has a special anatomical structure, ERE has different effects on liver tumors in different liver regions. The purpose of this work was to study the effect of off-site ERE on the quality of treatment plan for liver cancer under 1.5 T vertical magnetic field. The relationship between the quality of the plan and the number of subfields was observed to explore whether the new plan can compensate the dose disturbance caused by the static magnetic field.

## Method

### Patient selection

**Fig. 1:**
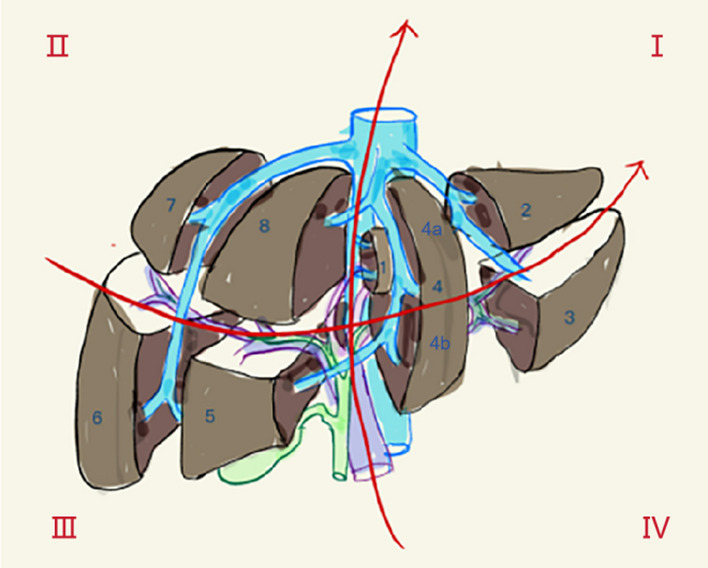
Schematic diagram of the anatomical division of the liver

**Fig. 2:**
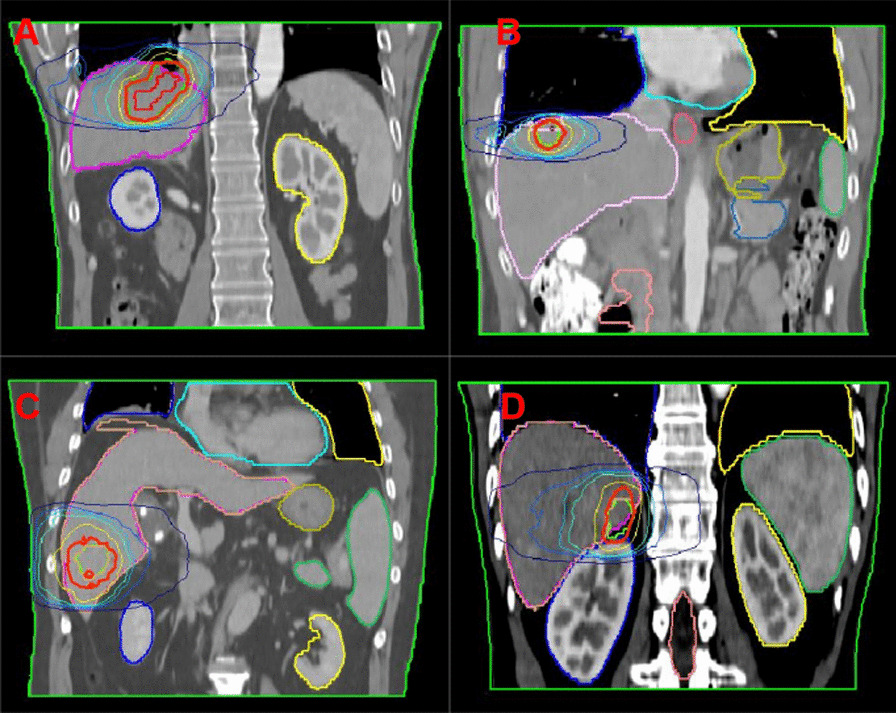
Schematic representation of liver masses in four patient quadrants. **A** Mass in the I quadrant; **B** mass in the II quadrant; **C** mass in the III quadrant; and **D** mass in the IV quadrant

A retrospective study was conducted with 62 patients who received RT for liver cancer in Shandong Cancer Hospital from February 2022 to February 2023. This study was approved by the Ethics Review Committee of Shandong Cancer Hospital (SDTHEC2022012021), and informed consent was obtained from all patients and their families. To better describe the relationship between the plan and the location of the mass, the liver was divided into four quadrants (Fig. 1). According to the distribution of the portal vein and hepatic vein in the liver, anatomical segmentation and lobulation was carried out, generally dividing the liver into 5 leaves and 8 segments. The liver was divided into four quadrants: 1, 2, 4a as the I quadrant, 7, 8 as the II quadrant, 5, 6 as the III quadrant, and 3, 4b as the IV quadrant (Fig. 2).

### MR-LINAC workflow

**Fig. 3:**
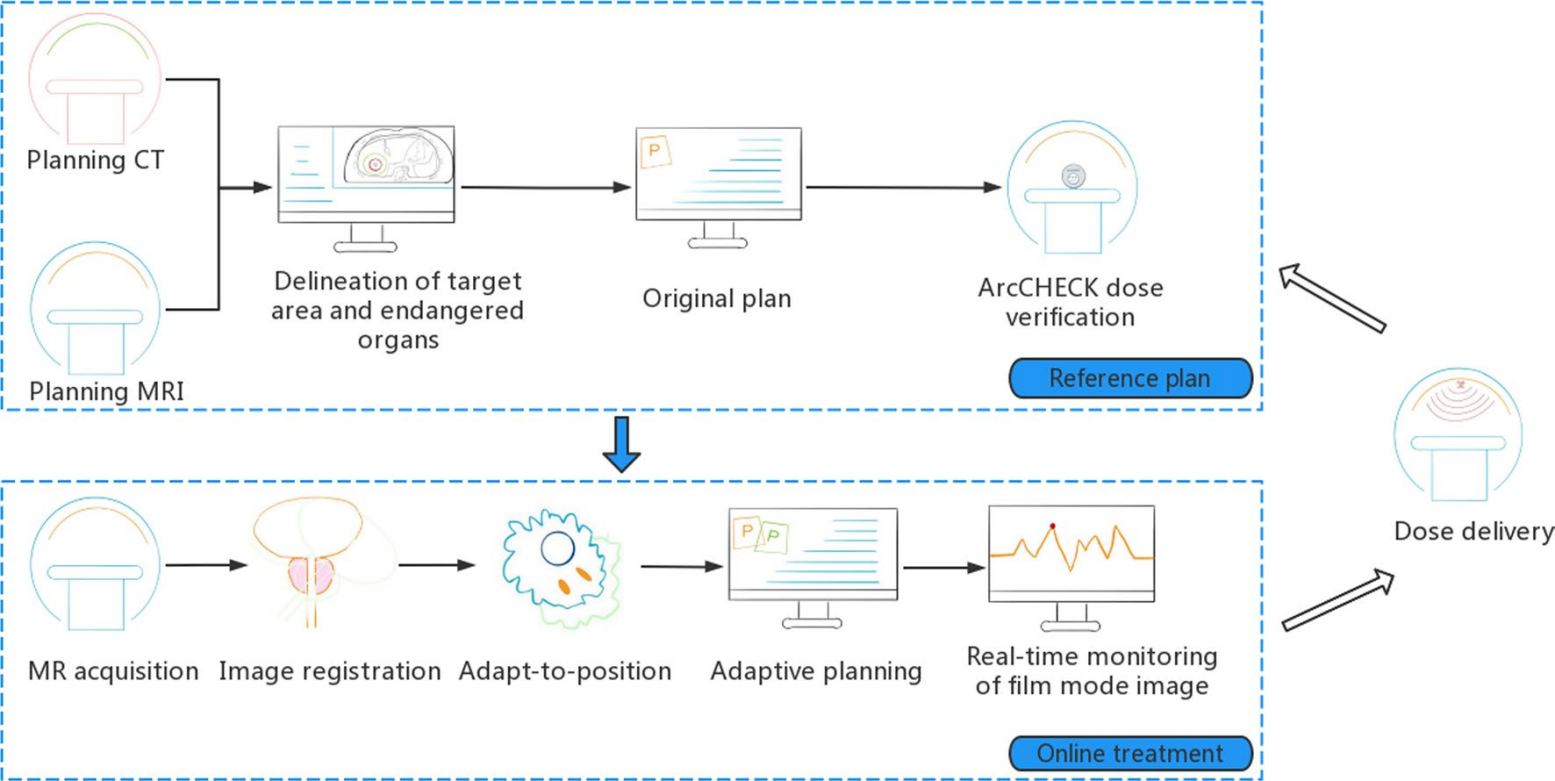
MRgRT workflow

Patients treated on the Elekta Unity MR-linac (Elekta Unity, Elekta AB, Stockholm, Sweden) received the Brilliance large-aperture CT (Royal Philips, Amsterdam, Netherlands) simulation with a tube voltage of 120 kV, a layer thickness of 1 mm, and a scan cycle of approximately 2 min. And using T2-weighted MRI (Siemens, Munich, Germany) scans (repetition time: 2100 ms, echo time: 205.585 ms, layer thickness: 1.2 mm) simulation on the same day. The supine position was fixed, the patient was in a state of free breathing, and the abdominal pressure band (Hymnsum, Shandong, China) was employed to lessen the effect of breathing. MR-LINAC can only carry out static intensity modulation scheme. (Fig. 3).

### Development and assessment of reference plans

Radiation oncologists with expertise in the treatment of liver cancer carried out the PTV and OARs drawings. The PTV was obtained by enlarging the gross target volume (GTV) of liver cancer patients treated using both machines by 3 mm, and the radiation dose was 95% of the PTV, V_10_ < 33.9 Gy. Table 1 lists the OARs restriction. The study did not include the lungs and hearts since the chest was not completely scanned in some participants. The prescribed dose for 32.3% of patients was 63 Gy/9 fractions/qd; for the remaining patients receiving conventional radiation, the prescribed doses were 19.4% 45 Gy/15 fractions/qd, 25.8% 40 Gy/8 fractions/qd, and 8.1% 50 Gy/25 fractions/qd. Based on the idea of equal distribution of the closest, the field is distributed. The dose rate of MR-LINAC is fixed at 400 MU/min.

**Table 1:** Planning objectives for organs at risk

Organs at risk	Dose constrain
Duodenum	V50 < 15%
Spinal cord	Max dose ≤ 40 Gy
Stomach	Max dose ≤ 40 Gy
Kidneys	V20 < 30%
Lung	D1500cc < 15 Gy
Heart	Max dose ≤ 42.5 Gy
Liver	Max dose < 45 Gy

*Max* maximum

The program evaluation was conducted by attending physicians and medical physicists using dose-volume histogram (DVH) indicators based on the same regimen. The uniformity and consistency of the dose in the target area were evaluated by the conformity index (CI) [19] and homogeneity index (HI) [20]. The dose distribution to the PTV and OARs was evaluated by average dose (D_mean_), minimum dose (D_min_), maximum dose (D_max_) and Vx (percentage of volume accepted xGy). This study is normalized and compared with the percentage of the target dose of DVH in the corresponding prescription dose because the patients' tumor sizes, shapes, and prescription doses varied.

HI and CI are defined as:

$${\text{HI}} = \frac{{{\text{D}}_{{98{\text{\% }}}} }}{{{\text{D}}_{{2{\text{\% }}}} }}$$

$${\text{CI}} = \frac{{{\text{V}}_{{{\text{T}},{\text{ref}}}} }}{{{\text{V}}_{{{\text{ref}}}} }}$$

D_2%_ and D_98%_ represent the minimum dose covering 2% and 98% of the target volume, respectively; V_T,ref_ refers to the target volume where the accepted dose is equal to or greater than the reference dose, and V_ref_ is the prescription equivalent dose volume.

### Optimization plan

On the basis of each patient's original customized intensity modulation reference plan (plan_1.5 T_), only the magnetic field setting was disabled, and the static intensity modulation plan without a magnetic field (plan_0 T_) was generated in order to compare the impact of magnetic field on the quality of the plan. Dosimetric parameter discrepancies between plans 1.5 T and 0 T were studied.

Four optimization strategies are developed to observe the impact of maximum subfield number and field density on fading magnetic field. The maximum subfield number is designed as "30, 60, 80" three critical values, and 15°uniform distribution of fields, according to clinical experience.

The first optimization scheme is designed to control only the static intensity modulation scheme (plan_30_), in which the maximum number of subfields is set to 30, which represents the low subfield number plan; the second optimization scheme is designed to control only the static intensity modulation scheme (plan_60_), in which the maximum number of subfields is set to 60, which represents the median subfield number plan; the second optimization scheme is designed to control only the static intensity modulation scheme (plan_80_), in which the maximum number of subfields is set to 80, which represents the high subfield number plan; the fourth is the multi-field static intensity modulation plan (plan_angle_), which raises the field angle to about 15°, with the exception of the direction in which the lead dose limit and OARs cannot be added. Table 2 displays the planning information for each plan. The dosimetric indices with a significant influence of the magnetic field are found by comparing the dosimetric characteristics between plan_1.5 T_ and plan_0 T_. Only these indexes are compared between plan_0 T_ and optimization plan to determine which optimization strategy is comparable to or superior to the non-magnetic field plan.

**Table 2:** Planning information for the optimization plan

	Plan_1.5 T_	Plan_0 T_	Plan_30_	Plan_60_	Plan_80_	Plan_angle_
Magnetic field	1.5 T	0 T	1.5 T	1.5 T	1.5 T	1.5 T
Max number of subfields	50	50	30	60	80	50
Number of beams	8 (5–10)	8 (5–10)	8 (5–10)	8 (5–10)	8 (5–10)	14 (13–16)

### Statistical analysis

IBM SPSS (Version 25.0) statistical software (IBM Corporation, Armonk, NY, USA) was used for statistical analysis. Prior to comparing the dose parameters, the data's normality was assessed using the Shapiro–Wilk test. The dosimetry parameters were compared by paired t test and Wilcoxon signed rank sum test.

## Results

### Patient and treatment characteristics

The median age of patients getting SBRT was 58.0 years, 72.3% of them were men, and 50% had primary liver cancer (Table 3). After the normal distribution test, the data is proved to be non-normal distribution, so the Wilcoxon signed rank method is used.

**Table 3:** Patient characteristics

	Quadrant I	Quadrant II	Quadrant III	Quadrant IV
Number of patients	13	20	17	12
PTV (cm^3^)	49.62 ± 64.28	51.58 ± 64.72	50.61 ± 65.13	51.88 ± 67.13

### Magnetic field influence

**Fig. 4:**
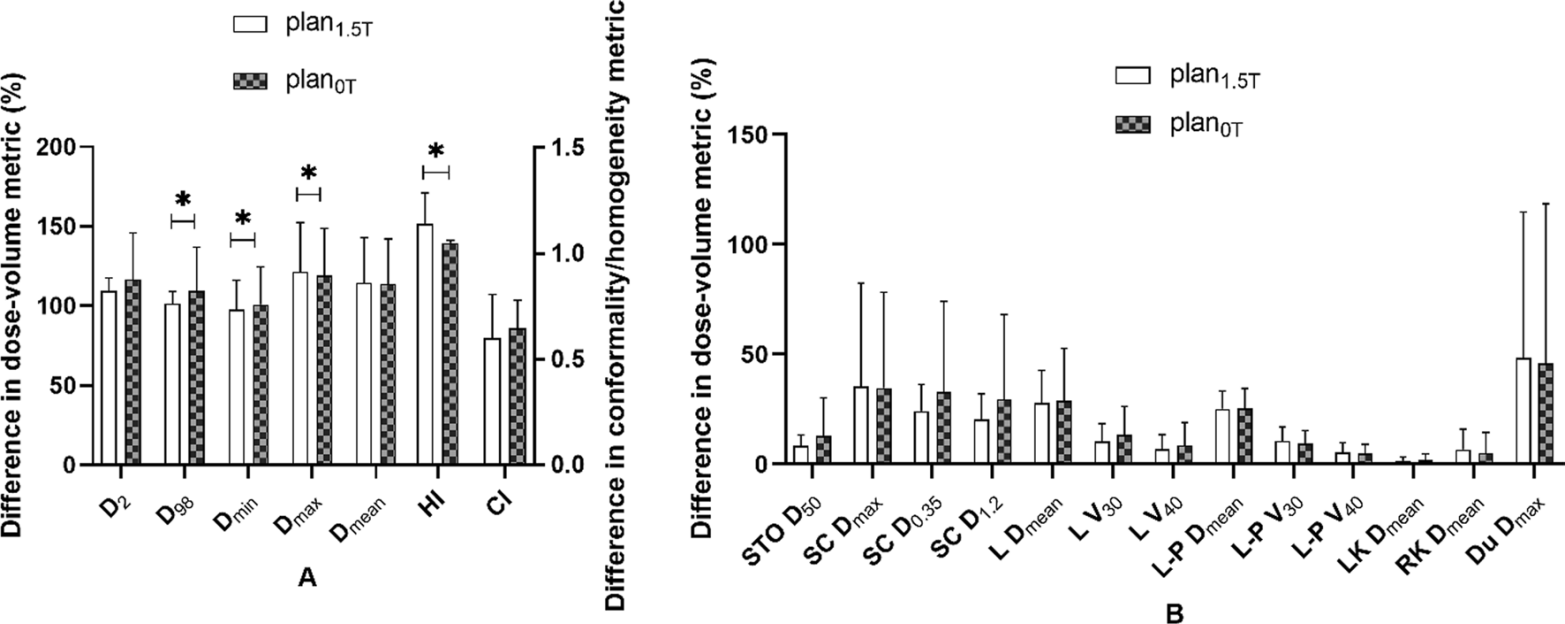
Comparison of dose parameters between plans with or without a magnetic field for the first quadrant. **A** Parameters of PTV, **B** parameters of OARs. *STO* stomach, *SC* spinal cord, *L* liver, *L-P* normal liver, *LK* left kidney, *RK* right kidney, *Du* duodenum

**Fig. 5:**
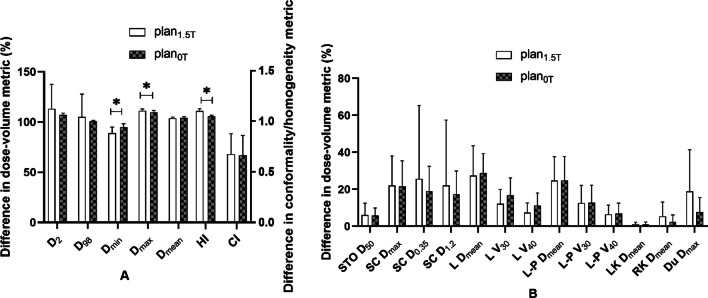
Comparison of dose parameters between plans with or without a magnetic field for the second quadrant. **A** Parameters of PTV, **B** parameters of OARs. *STO* stomach, *SC* spinal cord, *L* liver, *L-P* normal liver, *LK* left kidney, *RK* right kidney, *Du* duodenum

**Fig. 6:**
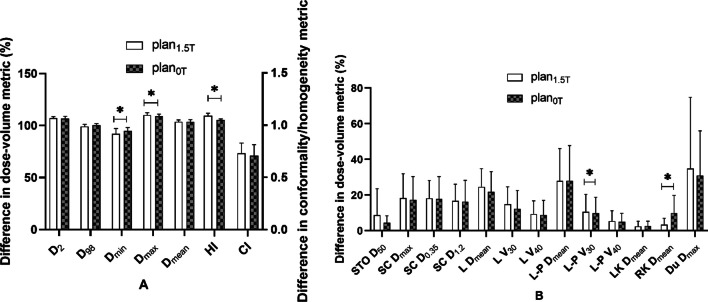
Comparison of dose parameters between plans with or without a magnetic field for the third quadrant. **A** Parameters of PTV, **B** parameters of OARs. *STO* stomach, *SC* spinal cord, *L* liver, *L-P* normal liver, *LK* left kidney, *RK* right kidney, *Du* duodenum

**Fig. 7:**
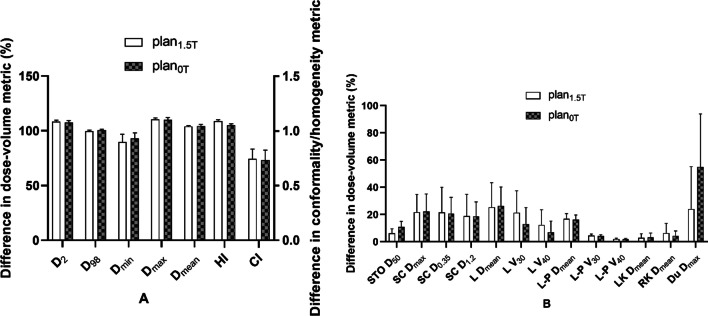
Comparison of dose parameters between plans with or without a magnetic field for the fourth quadrant. **A** Parameters of PTV, **B** parameters of OARs. *STO* stomach, *SC* spinal cord, *L* liver, *L-P* normal liver, *LK* left kidney, *RK* right kidney, *Du* duodenum

The RT plans of all patients in this study met the acceptance criteria. In Figs. 4, 5, 6 and 7, the distribution of doses to different organs among the plans are represented by column charts. The horizontal axis of the chart shows the DVH indicators and evaluation parameters. In the cases where DVHs is CI and HI, the vertical axis displays absolute values. In the cases where DVHs is Dx, the vertical axis displays the percentage of the prescription dose, and in the case of Vx, it displays the volume percentage that accepts the xGy value. Additionally, majority of the characteristics between groups showed variations. Asterisks were used to indicate the DVH parameters that differed significantly (*P* < 0.05). The Wilcoxon symbolic rank sum test is employed, and the parameters all fit the non-normal distribution.

The dose-volume difference between the treatment plans designed for 1.5 T and 0 T for the first quadrant is shown in Fig. 4. In the indicators involving PTV coverage and HI, there were statistically significant differences. For PTV, in the absence of a magnetic field, the indexes of most recalculated optimized plans were significantly improved, including an increase in PTV D_min_ (97.75 ± 17.55 vs. 100.96 ± 22.78)%, a decrease in D_max_ (121.48 ± 29.68 vs. 119.06 ± 28.52)%, and a decrease in HI (1.14 ± 0.14 vs. 1.05 ± 0.01). Some indexes also deteriorated significantly, including the increase in D_98_ (101.35 ± 7.42 vs. 109.35 ± 26.52)%. In the absence of a magnetic field, most of the indicators for OARs increased, but there were no significant differences.

The difference in dose-volume measurements between the treatment plans designed for 1.5 T and 0 T for the second quadrant is shown in Fig. 5. In the indicators involving PTV coverage and HI, there were statistically significant differences. For PTV, in the absence of a magnetic field, the indexes of all recalculated optimized plans were significantly improved, including an increase in PTV D_min_ (84.33 ± 19.74 vs. 89.96 ± 21.23)%, a decrease in D_max_ (105 ± 25.08 vs. 104.05 ± 24.86)%, and a decrease in HI (1.04 ± 0.25 vs. 0.99 ± 0.24). In the absence of a magnetic field, most of the indicators for OARs were reduced, but there were no significant differences.

The dose-volume difference between the treatment plans designed for 1.5 T and 0 T for the third quadrant is shown in Fig. 6. In the indicators involving PTV coverage and HI, there were statistically significant differences. For PTV, in the absence of a magnetic field, the indexes of most of the recalculated optimized plans were significantly improved, including decreases in PTV D_max_ (110.21 ± 2.22 vs. 102.31 ± 26)%, L-P V_30_ (10.66 ± 9.19 vs. 102.31 ± 3.22)% and HI (1.09 ± 0.02 vs. 0.98 ± 0.25). Some indexes also deteriorated significantly, including the decrease in PTV D_min_ (92.12 ± 4.92 vs. 89.1 ± 22.35)%. In the absence of a magnetic field, most of the indicators for OARs were reduced, but there were no significant differences.

The dose-volume difference between the treatment plans designed for 1.5 T and 0 T for the fourth quadrant is shown in Fig. 7. In the indicators involving PTV coverage and HI, there were statistically significant differences. For PTV, in the absence of a magnetic field, the indexes of most recalculated optimized plans were significantly improved, including an increase in PTV D_min_ (89.78 ± 6.72 vs. 93.04 ± 4.86)% and a decrease in HI (1.09 ± 0.01 vs. 1.05 ± 0.01). Some indexes also deteriorated significantly, including the increase in D_98_ (99.82 ± 0.82 vs. 100.54 ± 0.84)%. In the absence of a magnetic field, most of the indicators for OARs were reduced, but there were no significant differences.

### Optimization strategy

Among the dose parameters of plans with and without a magnetic field, there was a significant dose difference between the optimized treatment scheme designed for 1.5 T and the magnetic field-free plan, as shown in Table 2. In the first quadrant, plan_60_ and plan_80_ could achieve the same HI as plan0T, plan_30_ could achieve the same D_min_, and plan_60_ could achieve the same D_max_. In the second quadrant, plan_80_ could achieve the same HI and plan_80_ and plan_angle_ could achieve the same D_max_ with no magnetic field effect, and plan_80_ could achieve better D_min_ outcomes. In the third quadrant, plan_60_ and plan_80_ could achieve the same HI, plan_60_, plan_80_ and plan_angle_ could achieve the same D_max_, and the optimization strategy in L-P V_30_ all could achieve the effect of no magnetic field. In the fourth quadrant, plan60 and plan_80_ could achieve the effect of no magnetic field in terms of HI and D_min_ (Table 4). The radiation time and monitoring units of each optimized treatment regimen are shown in Table 5.

**Table 4:** Comparison of the dose differences between the optimized strategy and nonmagnetic field plan

	Plan_0 T_	Plan_30_	Plan_60_	Plan_80_	Plan_angle_
I quadrant					
HI	1.05 ± 0.01	1.08 ± 0.01 (0.027)	1.05 ± 0.01 (0.366)	1.05 ± 0.01 (0.527)	1.07 ± 0.03 (0.007)
D_min_(%)	100.96 ± 22.78	99.54 ± 35.2 (0.116)	98.34 ± 23.41 (0.006)	98.08 ± 22.4 (0.006)	97.82 ± 24.05 (0.013)
D_max_ (%)	119.06 ± 28.52	128.8 ± 40.96 (0.028)	120.11 ± 30.19 (0.087)	120.01 ± 29.65 (0.019)	121.04 ± 29.23 (0.019)
II quadrant					
HI	0.99 ± 0.24	1.08 ± 0.03 (0.002)	1.06 ± 0.02 (0.005)	1.05 ± 0.02 (0.142)	1.07 ± 0.02 (0.001)
D_min_ (%)	89.96 ± 21.23	86.6 ± 6.28 (0.002)	89.25 ± 5.29 (< 0.001)	90.01 ± 5.24 (0.001)	89.19 ± 5.61 (< 0.001)
D_max_ (%)	104.05 ± 24.86	111.35 ± 2.25 (0.002)	110.32 ± 1.79 (0.043)	110.1 ± 1.55 (0.184)	110.38 ± 1.36 (0.091)
III quadrant					
HI	0.98 ± 0.25	1.07 ± 0.02 (0.041)	1.05 ± 0.01 (0.837)	1.05 ± 0.01 (0.313)	1.09 ± 0.13 (0.006)
D_max_ (%)	102.31 ± 26	110.66 ± 1.92 (0.028)	109.48 ± 1.89 (0.002)	109.65 ± 1.99 (0.002)	109.82 ± 1.88 (0.031)
L-P V30 (%)	5.81 ± 3.22	10.77 ± 8.24 (0.080)	10.65 ± 9.6 (0.110)	7.82 ± 4.18 (0.753)	9.54 ± 6.98 (0.499)
IV quadrant					
HI	1.05 ± 0.01	1.08 ± 0.01 (0.007)	1.06 ± 0.01 (0.132)	1.06 ± 0.01 (0.180)	1.06 ± 0.01 (0.002)
D_min_ (%)	93.04 ± 4.86	85.34 ± 10.26 (0.008)	90.17 ± 6.65 (0.060)	92.09 ± 3.78 (0.091)	90.31 ± 5.62 (0.015)

**Table 5:** Parameter information related to optimization plan

	Total MU	Total time (min)
Plan30	74,265.77	283.5
Plan60	79,828.038	338.2
Plan80	81,118.94	388.3
Planangle	72,467.46	330.2

## Discussion

By comparing the simulated dose distributions of plans without a magnetic field and those with a 1.5 T magnetic field, the effect of the magnetic field was evaluated. The results of this study show that the existence of a magnetic field leads to a systematic difference in dose exposure between the target and OARs. Theoretically, the dose transfer guided by MR is inevitably affected by the permanent magnetic field [21]. The magnetic field-induced change is caused by the change in dose deposition in the tissue, which affects the trajectory of secondary electrons that return to the tissue surface at the air-tissue interface [22], that is, the so-called ERE, causing the beam dose deposition nucleus to become obviously asymmetric in the direction perpendicular to the 1.5 T magnetic field [10]. Compared with the nonmagnetic field plan, the original plan with a magnetic field for each quadrant, the dose uniformity and the minimum and maximum doses to the target area were worse, and the dose received by the OARs was increased. It will worsen the target dose's inhomogeneity and increase or decrease the interfacial dose under the concurrent influence of tissue inhomogeneity and ERE [23]. This conclusion is similar to that reported by Nedaie HA et al. [24]. The results also show that in the treatment plans for liver cancer, the effect of a magnetic field on OARs is more stable than that on the target area. It might be because the target area will be impacted by the magnetic field's superposition and receive more beams than the OARs. However, in the first quadrant, in the absence of a magnetic field, the dose to the OARs is relatively increased, which may be because compared with other regions, the first quadrant’s tumors adjacent to more OARs and represents a more complex scattering environment. The heart, lungs, esophagus, and other significant hollow organs are examples. The gas in the treatment ray path may make dosage distortion worse with ERE [25], and the small sample size may lead to inconsistent results.

In the second part of the study, it was found that when designing plan for liver tumors in the first quadrant, the total number of control subfields should be 60; the total number of liver tumor planning control subfields in the second quadrant should be 80; the total number of liver tumor planning control subfields in the third quadrant and the fourth quadrant should be 60 or 80, the dosage effect of the magnetic field on the patients can be greatly reduced or even completely eliminated via plan adjustment. Normally, the magnetic field would impact on the fluence of the photon [26]. This fluence would depend on the prescription dose and number of subfields. The MLC field strength contains numerous subfield control points, which define the MLC shape [27]. The greater the number of control points is, the higher the beam intensity level [28]. Although the target dose is high, there are more hot areas, thus it has two sides. The findings of this study indicate that the maximum sub-field number should be customized for various liver tumor subtypes since it cannot be generalized. The blade of the MLC experiences continuous movement in RT, and the field intensity is adjusted to emit the beam to change the quality of the plan [29]. The results of this study show that increasing the beam dose has minimal effect on counteracting the magnetic field and even led to an increase in the dose to OARs due to the encryption of the field, which may be due to its limited field size and complex irradiation trajectory and low the irradiation efficiency [10]. However, the use of the relative beam itself counteracts the uneven dose caused by ERE and limits the freedom of IMRT planning. Raaijmakers et al. proposed that when irradiating an inhomogeneous anatomical target in the presence of a magnetic field, a relative beam is not necessary [30]. Overencrypting the field will degrade the quality of the plan.

In this study, it was recognized that controlling the total number of different subfields in different quadrants could result in better RT plans, which can largely offset the dose changes caused by magnetic fields. In addition, with extensions in treatment time and increases in hop count, Anatomical environment movement may somewhat increase, and the subfield number should be set to a feasible choice to ensure adequate dose distribution while keeping the treatment time as short as possible. According to the findings, plan_80_'s radiation schedule was 50 min longer than plan_60_'s, and each patient received an insignificant one-minute-long treatment on average. Normal tissue and target volume optimization constraints follow individual/institutional preferences, which may lead to different results from this study. Nevertheless, the optimization plan currently used reflects the goal of achieving optimal target coverage and dose uniformity based on the agency's experience.

This study has several limitations. First, the sample size is relatively small, and in future studies, our goal is to include more liver SBRT cases to further verify the model. The results would be more persuasive if validated with prospective data in a clinical context. Second, previous RT records were not accounted for in this study. The changes in liver properties caused by previous liver treatment may affect the dose distribution. Third, compared with the traditional linear accelerator, the dose rate of MR-LINAC is lower, resulting in longer treatment time. However, the similarities and differences between the two are not clear in the existing research, and further exploration is needed in the follow-up research.

## Conclusion

In summary, the effect of a 1.5 T transverse magnetic field on the target dose was more significant than that on the dose to OARs in patients with liver cancer. By optimizing the max total number of subfields of liver masses in different quadrants, the influence of the magnetic field can be greatly reduced or even eliminated, thus avoiding deterioration of the overall plan quality.
